# The concentration of tumor necrosis factor-α determines its protective or damaging effect on liver injury by regulating Yap activity

**DOI:** 10.1038/s41419-020-2264-z

**Published:** 2020-01-27

**Authors:** Shanmin Zhao, Jinghua Jiang, Yingying Jing, Wenting Liu, Xue Yang, Xiaojuan Hou, Lu Gao, Lixin Wei

**Affiliations:** 1 Tumor Immunology and Gene Therapy Center, Third Affiliated Hospital of Second Military Medical University, Shanghai, 200438 China; 2 Laboratory Animal Center of Second Military Medical University, Shanghai, 200433 China

**Keywords:** Liver diseases, Experimental models of disease

## Abstract

Previous studies have shown that tumor necrosis factor (TNF)-α is a mediator of hepatotoxicity in liver injury. Moreover, TNF-α has also been reported to have a protective effect in liver regeneration, yet the function of TNF-α during liver injury remains controversial. Here, we report that the concentration of TNF-α determines its functions. High concentrations of TNF-α could aggravate LPS-induced liver injury. However, the TNF-α level was unchanged during APAP-induced liver injury, which exerted a protective effect. We expected that the concentration of TNF-α may affect its function. To test this hypothesis, TNF-α^−/−^ rats or hepatocyte cells were treated with different concentrations of TNF-α. We found low TNF-α could reduce the levels of ALT and AST in the plasma of TNF-α^−/−^ rats and promote the proliferation of hepatocyte cells. However, the levels of ALT and AST increased gradually with increasing TNF-α concentration after reaching the lowest value. Moreover, we showed that TNF-α affects the cell proliferation and cell death of hepatocytes by regulating Yap activity. Low TNF-α promoted Yap1 nuclear translocation, triggering the proliferation of hepatocytes. However, high TNF-α triggered the phosphorylation and inactivation of Yap1, preventing its nuclear import and consequently promoting cell death. Collectively, our findings provide novel evidence that the concentration of TNF-α is an important factor affecting its function in liver injury, which may provide a reference for the clinical treatment of liver injury.

## Introduction

Tumor necrosis factor-α (TNF-α) is a pleiotropic cytokine in disease pathogenesis such as liver injury, which governs development of the immune system, cell survival signaling pathways, proliferation, and regulates metabolic processes^1–3^. Many studies have focused on the role of TNF-α in the occurrence and development of liver injury. It has been also reported that TNF-α plays a significant role by inducing hepatocyte apoptosis, which mediates hepatotoxicity in lipopolysaccharide (LPS)- or concanavalin A-induced liver injury^4–6^. Moreover, TNF-α is important for liver regeneration and tissue repair following acetaminophen (APAP)-induced hepatotoxicity^7^. Grivennikov et al.^8^ even showed TNF-α could be either protective, as in host defense, or deleterious, as in autoimmunity or toxic shock. Thus TNF-α has dual function in liver injury, either aggravating or alleviating injury, which presents a challenge for designing treatments to prevent liver injury.

Studies have shown that liver injury induced by different causes, such as viruses, bacteria, etc., has relatively specific inflammatory microenvironment characteristics, which could induce rapid immune response, infiltration of inflammatory cells, and production of inflammatory factors in the liver, thus destroying the immune balance in the liver and inducing a series of liver pathological processes. However, some drugs or chemicals (APAP, CCl_4_, etc.) induce hepatic injury, which starts with hepatocytes^9^. A large number of intermediate metabolites trigger hepatocyte necrosis through a series of metabolic reactions, release intracellular contents, and subsequently induce inflammation^10^. Therefore, the concentration of TNF-α in the liver differs depending on the cause of liver injury, and whether this affects the function of TNF-α is still unclear.

Yap is a critical component of the Hippo pathway, which regulates the proper size of organs through a balance of cell growth and cell death^11–13^. When Hippo signaling is active, Yap is phosphorylated and restricted to the cytoskeleton^14^. Loss of phosphorylation, whether by decreased kinase activity or through increased phosphatase activity, is associated with nuclear localization of Yap and the subsequent activation of downstream proliferative and anti-apoptotic gene programs^11,15^. Liu et al.^16^ showed that activation of Yap attenuates hepatic damage and fibrosis in liver ischemia-reperfusion injury. Deletion of Yap in the liver leads to defects in both hepatocyte survival and biliary epithelial cell development^17^. However, the role of Yap activity in the determination of cell fate induced by TNF-α remains unknown.

In this study, we confirmed that TNF-α aggravated acute liver injury induced by lipopolysaccharide (LPS), but exerted a protective effect on APAP-induced liver injury. Further results showed that the concentration of TNF-α determined its protective or damaging effect on liver injury. Moreover, we showed that TNF-α functioned as an important factor in regulating the proliferation and cell death of hepatocytes via Yap1 activity.

## Results

### TNF-α knockout alleviates LPS-induced liver injury but aggravates APAP-induced liver injury

**Fig. 1:**
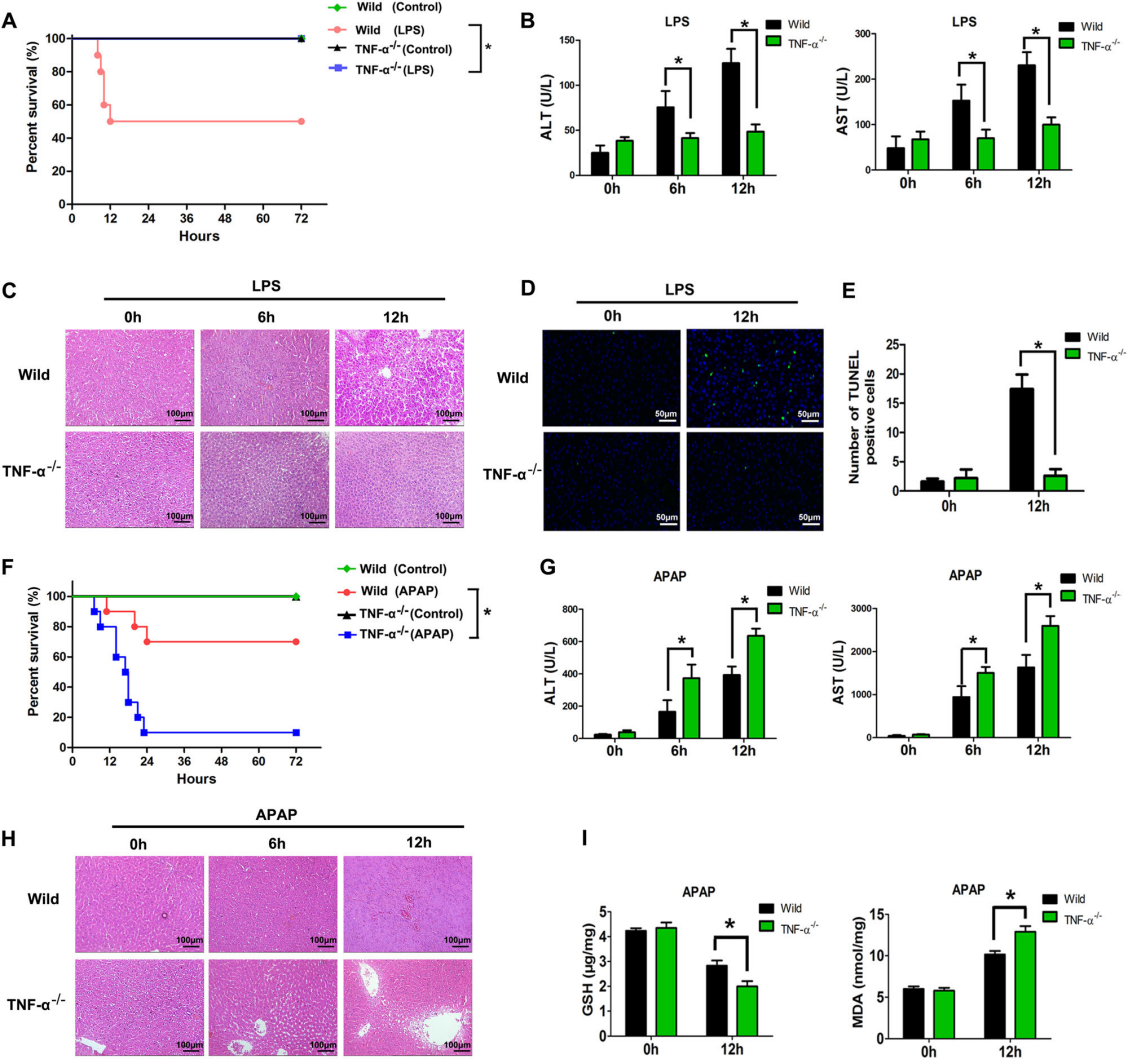
Deletion of TNF-α could resist LPS-induced liver injury, but aggravates APAP-induced liver injury in rats. **a** The survival rate of WT rats and TNF-α^−/−^ rats after LPS injection (*n* = 10 for each group). **b** Plasma samples were harvested at 0, 6, and 12 h after LPS injection for the measurement ALT and AST in rats (*n* = 4 for each time point). **c** H&E staining of liver sections from WT rats and TNF-α^−/−^ rats at 0, 6, and 12 h after LPS injection (magnification ×200). **d** TUNEL assay of liver sections from WT rats and TNF-α^−/−^ rats treated with LPS (magnification ×200). **e** Quantitative analysis of TUNEL-positive cells. **f** The survival rate of WT rats and TNF-α^−/−^ rats after APAP injection (*n* = 10 for each group). **g** Plasma samples were harvested at 0, 6, and 12 h after APAP injection for the measurement ALT and AST in rats (*n* = 4 for each time point). **h** H&E staining of liver sections from WT rats and TNF-α^−/−^ rats at 0, 6, and 12 h after APAP injection (magnification ×200). **i** GSH and MDA levels in the livers of WT rats and TNF-α^−/−^ rats 12 h after APAP injection (*n* = 3 for each time point). Data are expressed as the mean ± SD. *p < 0.05. Data are representative of at least three independent experiments.

Fifty percent of wild-type (WT) rats died within 12 h after challenge with 10 mg/kg LPS, but none of the challenged TNF-α^−/−^ rats died (Fig. 1a). Higher levels of plasma ALT and AST were observed in WT rats than in TNF-α^−/−^ rats after LPS administration (Fig. 1b). Histological examination of the liver showed significant hepatocyte death in WT rats after LPS administration but minimal changes in TNF-α^−/−^ rats (Fig. 1c). Furthermore, WT rats showed an increase in the number of terminal dUTP nick-end labeling (TUNEL)-positive apoptotic cells in the liver, while such cells were scarcely observed in TNF-α^−/−^ rats (Fig. 1d, e). TNF-α is known to be mainly produced by Kupffer cells^18,19^. We then depleted Kupffer cells by intravenous injection of GdCl_3_ (Fig. S2A). As shown in Fig. S2B, the depletion of Kupffer cells resulted in a reduction in TNF-α secretion in the plasma. The survival rate in the GdCl_3_ + LPS group was remarkably higher than that in the LPS-induced injury group (Fig. S2C). The levels of ALT and AST were decreased compared with those of the LPS-induced injury group (Fig. S2D). These data suggested that liver injury induced by LPS was mediated by TNF-α. TNF-α had damaging effect on LPS-induced liver injury.

We then compared APAP-induced hepatotoxicity in WT and TNF-α^−/−^ rats. The results showed that the survival rate of TNF-α^−/−^ rats was lower than that of WT rats after APAP injection (Fig. 1f), which was supported by increased levels of plasma ALT and AST (Fig. 1g) and an obvious aggravation of liver necrosis (Fig. 1h). Hepatic oxidative stress is regarded as the central mediator of APAP-induced acute liver damage^20,21^. We also found that TNF-α knockout worsened the oxidative stress by reducing the expression of hepatic GSH and enhancing MDA levels in liver tissues (Fig. 1i). These data suggested that TNF-α^−/−^ rats showed significantly increased sensitivity to the hepatotoxic effects of APAP than WT rats. TNF-α exerted a protective effect on APAP-induced liver injury.

### The concentration of TNF-α determines its effect on liver injury

**Fig. 2:**
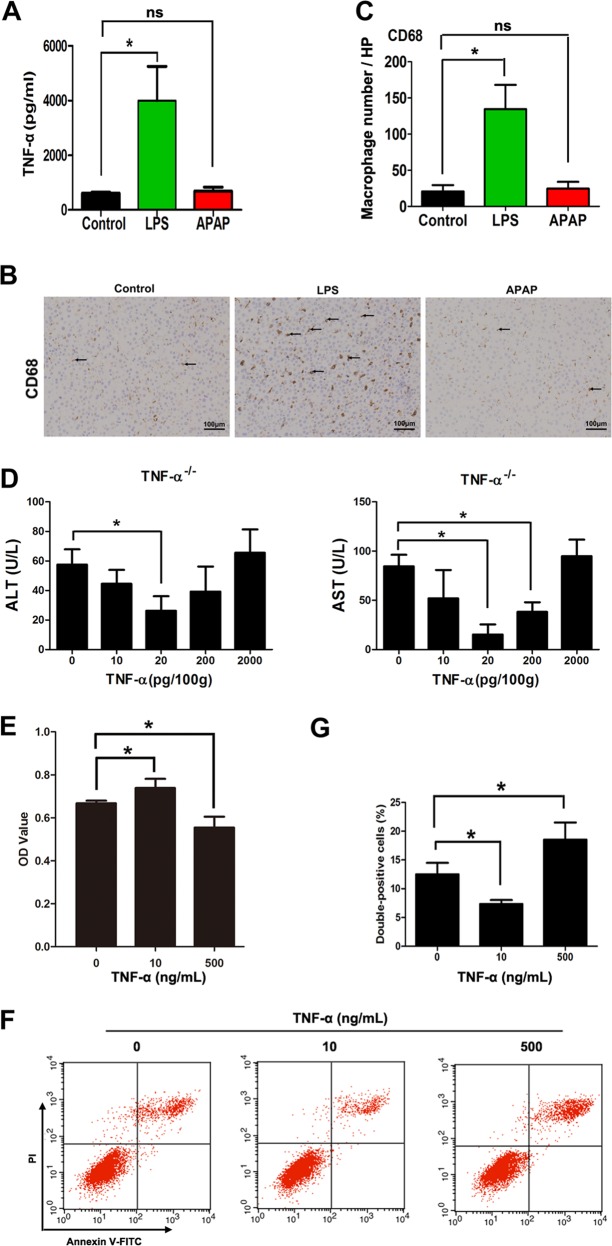
Different concentrations of TNF-α have opposite effects on cell fate. **a** TNF-α secretion in the livers of WT rats injected with LPS or APAP (*n* = 4 for each group). **b**, **c** Immunohistochemical staining of CD68^+^ Kupffer cells and numbers of positive cells in the livers of WT rats injected with LPS or APAP. Four liver sections were randomly selected from each group and used for immunohistochemistry analysis. Arrows indicates the CD68^+^ Kupffer cells. **d** Plasma ALT and AST levels in TNF-α^−/−^ rats 12 h after injection of different concentrations of TNF-α (*n* = 4 for each group). **e** CCK-8 counts of BRL cells treated with different doses of TNF-α for 6 h (*n* = 6 for each group). **f**, **g** Cell death detection of BRL rat hepatocytes treated with different doses of TNF-α for 6 h using an Annexin V-FITC/PI double staining flow cytometry assay (*n* = 3 for each group). Data are expressed as the mean ± SD. *p < 0.05. Data are representative of at least three independent experiments.

Liver injury caused by different factors showed different immunity reaction. We measured TNF-α levels in the liver at the early stage of LPS- and APAP-induced liver injury. The results showed that TNF-α secretion in the liver of WT rats increased significantly for 6 h after LPS injection (Fig. 2a). However, there was no significant difference in TNF-α levels at 6 h after APAP injection compared with the control group (Fig. 2a). It was showed that continuous LPS exposure could lead to the activation of Kupffer cells, etc^8^. A marker of macrophage CD68 were determined between two models. The result showed increased numbers of CD68^+^ cells in the livers of the LPS-induced liver injury group but no significant differences in the APAP-induced liver injury group (Fig. 2b, c). These results showed that a high TNF-α concentration was present in the liver upon LPS-induced injury, but a low TNF-α concentration was present upon APAP-induced injury.

To investigate the role of the concentration of TNF-α in acute liver injury, we tested the impact of different concentrations of TNF-α on hepatocytes. Different concentrations of TNF-α were injected into TNF-α^−/−^ rats. The results showed that low concentrations of TNF-α (0–20 pg/100 g) could reduce the levels of ALT and AST in the plasma. However, the levels of ALT and AST elevated with increasing TNF-α concentrations (higher than 20 pg/100 g; Fig. 2d). Furthermore, a low concentration of TNF-α (10 ng/mL) could promote the proliferation of BRL cells (Fig. 2e), while a high concentration of TNF-α (500 ng/mL) could increase the rate of Annexin V-FITC/PI double positive cells (Fig. 2f, g).

### The protective or damaging effects of TNF-α are mediated by TNFR1

**Fig. 3:**
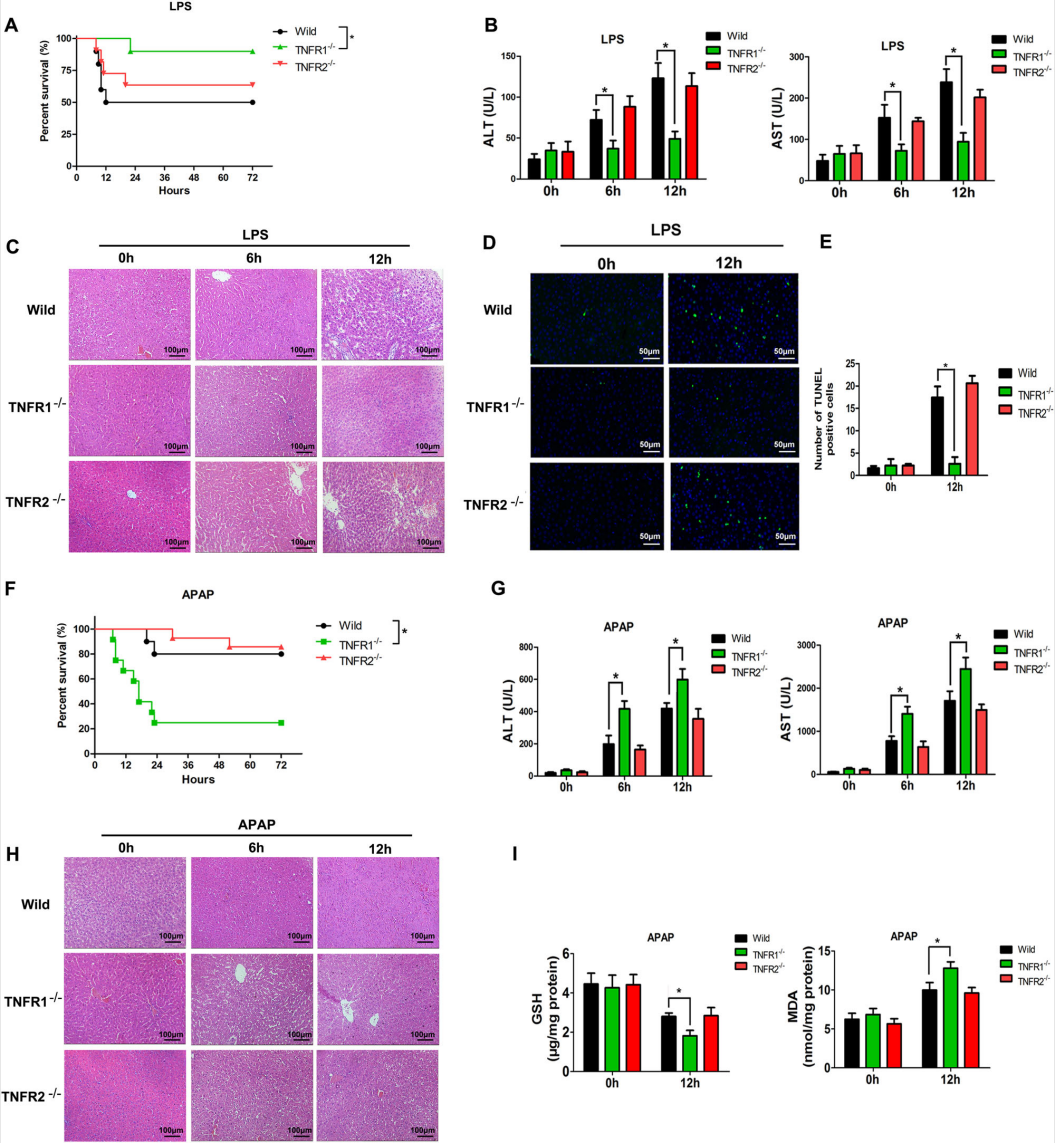
TNF-α involves in LPS-induced liver injury or alleviates APAP-induced liver injury through TNFR1. **a** The survival rate of WT rats, TNFR1^−/−^ rats and TNFR2^−/−^ rats after LPS injection (*n* = 10 for each group). **b** The levels of ALT and AST in the plasma were determined at 0, 6, and 12 h after LPS injection (*n* = 4 for each time point). **c** H&E staining of liver sections from WT rats, TNFR1^−/−^ rats and TNFR2^−/−^ rats at 0, 6, and 12 h after LPS injection (magnification ×200). **d** TUNEL assay of liver sections from WT rats, TNFR1^−/−^ rats and TNFR2^−/−^ rats treated with LPS (magnification ×200). **e** Quantitative analysis of TUNEL-positive cells. **f** The survival rates of WT rats, TNFR1^−/−^ rats and TNFR2^−/−^ rats after APAP injection (*n* = 10, 12, and 14, respectively). **g** Plasma samples were harvested at 0, 6, and 12 h after APAP injection for the measurement ALT and AST in rats (*n* = 4 for each time point). **h** H&E staining of liver sections from WT rats, TNFR1^−/−^ rats and TNFR2^−/−^ rats at 0, 6, and 12 h after APAP injection (magnification ×200). **i** GSH and MDA levels in the livers of WT rats, TNFR1^−/−^ rats and TNFR2^−/−^ rats 12 h after APAP injection (*n* = 3 for each time point). Data are expressed as the mean ± SD. *p < 0.05. Data are representative of at least three independent experiments.

TNF-α mediates its biological effects through two different receptors, which is TNFRSF1A (TNFR1) and TNFRSF1B (TNFR2). TNFR1^−/−^ rats had significantly increased survival rate after LPS-induced liver injury compared with WT rats (Fig. 3a), which was supported by the attenuated levels of plasma ALT and AST (Fig. 3b) and an obvious reduction in hepatic cell death (Fig. 3c–e). Moreover, an increase in the survival rate of TNFR1^−/−^ rats treated with APAP compared with WT rats was observed (Fig. 3f), accompanied by a significant increase in the levels of plasma ALT and AST (Fig. 3g). Morphologically, hepatic injury was also greater in TNFR1^−/−^ rats compared with WT rats (Fig. 3h). Also, TNFR1 knockout reduced the expression of hepatic GSH and enhanced MDA levels in liver tissues (Fig. 3i). Immunofluorescence staining showed that the expression of TNFR1 receptor is higher in the cytoplasm of BRL cells than that of TNFR2 receptor (Fig. S3).

**Fig. 4:**
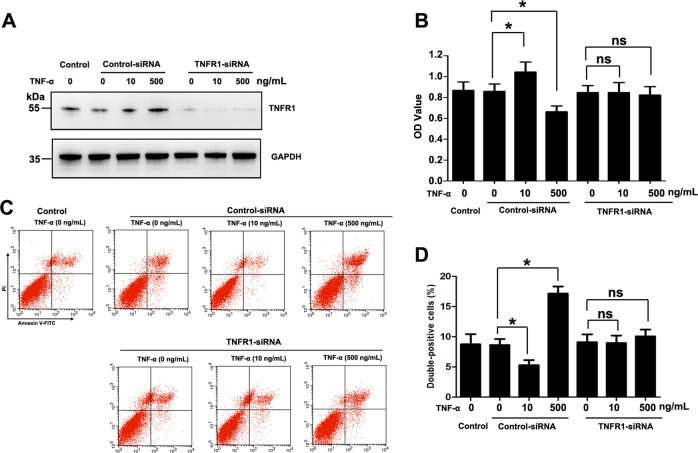
The proliferation and cell death effects of TNF-α are mediated by TNFR1. **a** Western blot analysis of TNFR1 in BRL cells transfected with control-siRNA or TNFR1-siRNA. **b** BRL cells were transfected with control-siRNA or transfected with TNFR1-siRNA for 12 h, and then treated with different concentrations of TNF-α (0, 10, or 500 ng/mL) for 6 h. Cell viability was determined using CCK-8 counts (*n* = 6 for each group). **c**, **d** Apoptotic cells were assayed using an Annexin V-FITC/PI double staining flow cytometry assay (*n* = 3 for each group). The cells Data are expressed as the mean ± SD. *p < 0.05. Data are representative of at least three independent experiments.

Next, we used a siRNA against TNFR1 to assess the role of TNFR1 in BRL cells treated with different concentrations of TNF-α. Western blotting was performed to analyze the expression level of TNFR1 protein. The results showed that less TNFR1 protein was detected in the siTNFR1-transfected BRL cells after transfection for 12 h (Fig. 4a). In the presence of TNFR1, low concentration of TNF-α could promote proliferation of BRL cells, and high concentration of TNF-α leads to cell death. However, proliferation of cells induced by a low concentration of TNF-α alleviated by silencing of TNFR1 (Fig. 4b) and the high concentration of TNF-α induced death of cells (Annexin V-FITC/PI double positive cells) were also inhibited by silencing of TNFR1 (Fig. 4c, d). These data indicated that the protective or damaging effects of TNF-α were mediated via the same receptor-TNFR1.

### Low TNF-α activates Yap while high TNF-α inhibits Yap activation

**Fig. 5:**
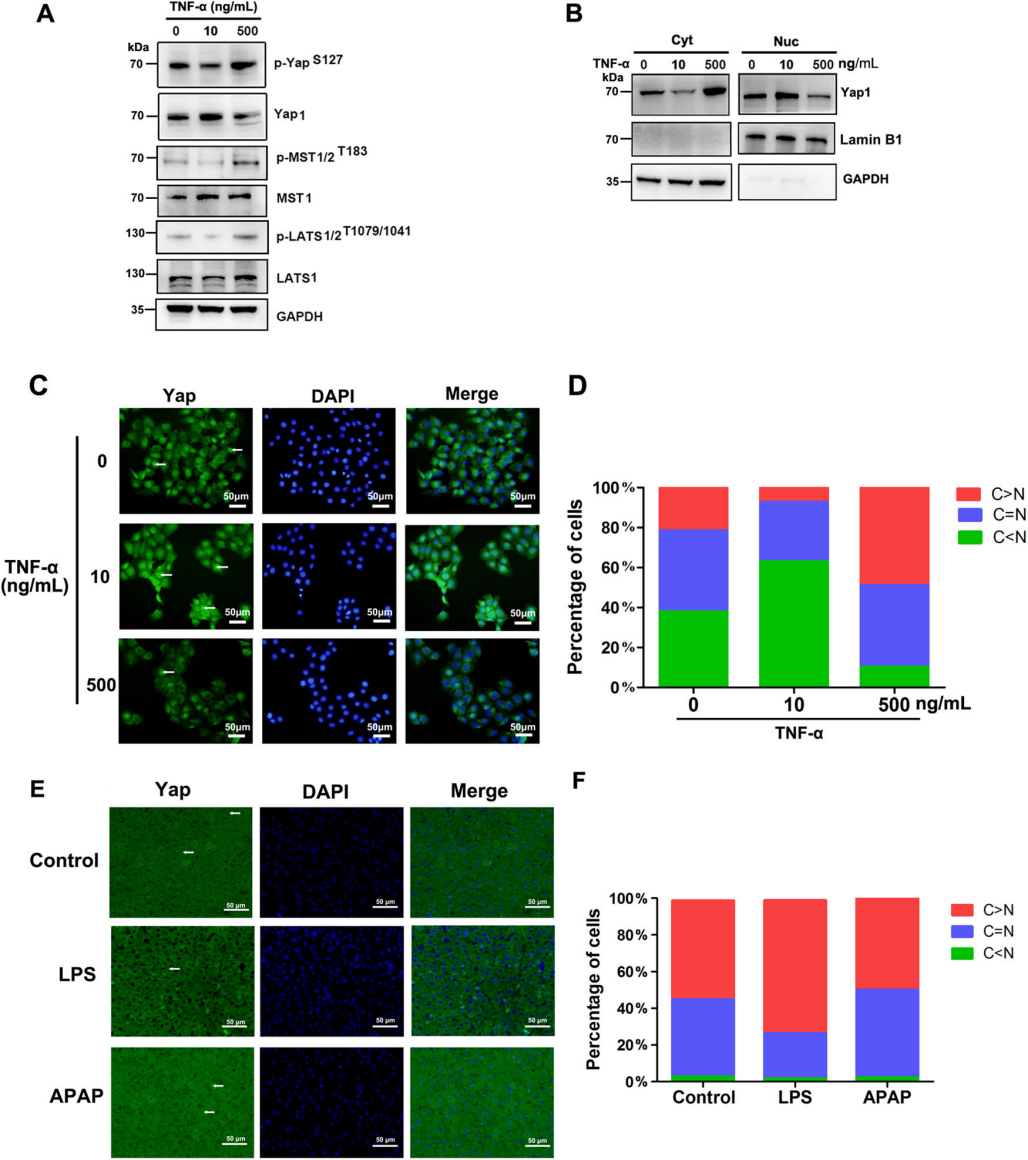
Different concentrations of TNF-α have different effects on the Yap activity of hepatocytes. **a** Western blotting with the indicated antibodies of BRL cells treated with different doses of TNF-α for 6 h. **b** Immunoblot analysis of Yap1 in cytoplasmic (Cyt) and nuclear (Nuc) fractions of BRL cells after different treatments. **c** Immunofluorescence of the distribution of Yap1 in BRL cells after different treatments. Endogenous Yap1 (green) and nuclei (blue) were stained with specific antibody and DAPI, respectively. Arrowheads depict nuclear Yap1 expression in BRL cells. **d** Quantifications of Yap1 subcellular localization from at least 100 randomly selected cells in **c**. **e** Immunohistochemistry for Yap1 localization and expression in livers from rats treated with LPS or APAP and in normal rat liver. Arrowheads depict nuclear Yap1 expression in hepatocytes. **f** Quantifications of Yap1 subcellular localization from at least 200 randomly selected cells in **e**. C cytoplasm, N nucleus. Data are representative of at least three independent experiments.

The expression of Yap1 in BRL cells treated with a low concentration of TNF-α (10 ng/mL) was increased, and the expression of p-MST1/2^T183^, p-LATS1/2^T1079/1041^ and p-Yap^S127^ was decreased (Fig. 5a). The expression of Yap1 in the cytoplasm was decreased and that of Yap1 in the nucleus was increased after treatment with a low concentration of TNF-α (Fig. 5b). The fluorescence intensity of Yap1 in the nucleus was increased (Fig. 5c). These results suggested that a low concentration of TNF-α promoted Yap activation. However, the expression of Yap1 in BRL cells treated with a high concentration of TNF-α (500 ng/mL) was decreased and the expression of p-Yap^S127^, p-MST1/2^T183^, and p-LATS1/2^T1079/1041^ were increased (Fig. 5a). In addition, the expression of Yap1 in the nucleus was decreased (Fig. 5b), Immunofluorescence staining showed that addition of 10 ng/mL TNF-α to the culture medium increased nuclear Yap localization compared to control group, but addition of 500 ng/mL TNF-α decreased the nuclear localization of endogenous Yap compared to control group (Fig. 5c, d). In LPS treated group, the intensity of Yap fluorescence in the hepatocyte nuclei were lower than that of control group and APAP group. (Fig. 5e, f). Taken together, these results indicated that a high concentration of TNF-α activated the Hippo signaling pathway and deactivated Yap phosphorylation.

### TNF-α affects the proliferation and cell death of hepatocytes by regulating Yap1 activity

**Fig. 6:**
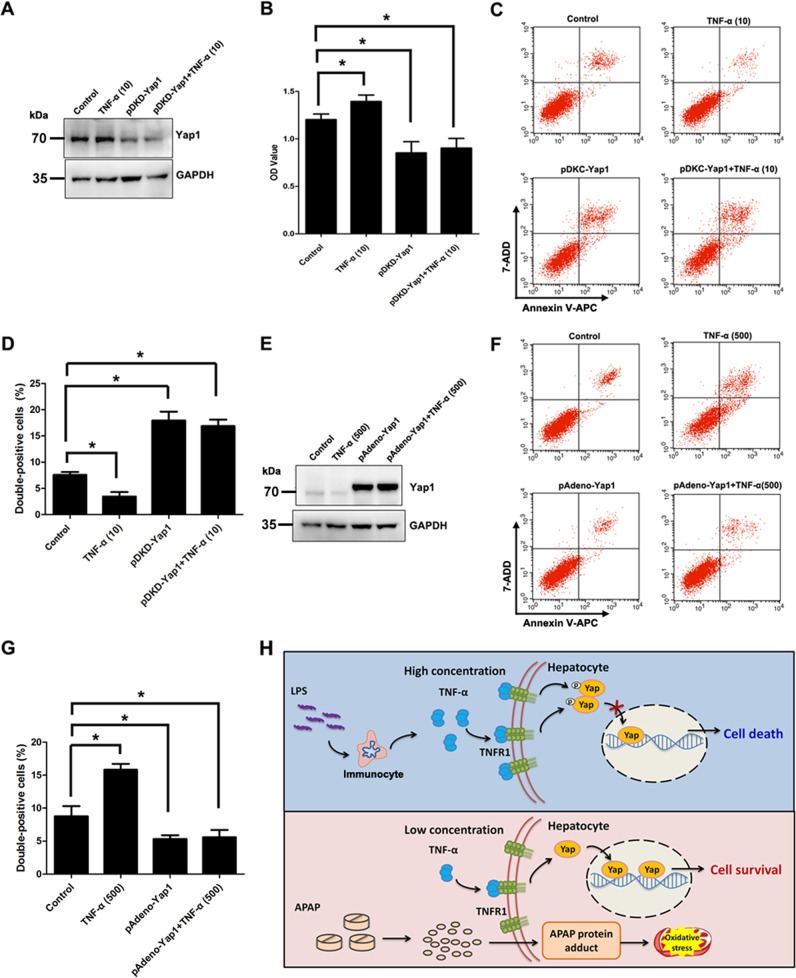
TNF-α regulates the proliferation and cell death of hepatocytes through the Yap pathway. **a** Western blotting analysis of the knockdown efficiencies of adenovirus-mediated shRNAs targeting Yap1 (pDKD-Yap1) for 12 h. **b** CCK-8 was used to evaluate the proliferation capacity of pDKD-Yap1 transfected BRL cells treated with TNF-α for 6 h (*n* = 5). **c**, **d** Flow cytometry analysis of transfected BRL cells treated with 10 ng/mL TNF-α for 6 h using an Annexin V-APC/7-AAD double staining flow cytometry assay (*n* = 3 for each group). **e** Western blotting analysis of the overexpression efficiencies of adenovirus-mediated pAdeno-Yap1 vector (pAdeno-Yap1) for 12 h. **f**, **g** Flow cytometry analysis of pAdeno-Yap1 transfected BRL cells treated with 500 ng/mL TNF-α for 6 h using an Annexin V-APC/7-AAD double staining flow cytometry assay (*n* = 3 for each group). **h** A proposed working model for the interplay of the TNF-α/TNFR1 pathway and Yap activity for cell fate determination. Data are expressed as the mean ± SD. *p < 0.05. Data are representative of at least three independent experiments.

We next sought to assess the role of Yap activity in cell fate determination during TNF-α treatment. We used gain- and loss-of-function experiments to evaluate the effect of Yap1. Western blotting assays showed that the expression of Yap1 protein was significantly decreased compared to that in the Yap1 control group (Fig. 6a). It was demonstrated that downregulation of Yap in BRL cells inhibited cell proliferation induced by a low concentration of TNF-α (Fig. 6b) and promoted cell death (Annexin V-APC/7-AAD double positive cells) (Fig. 6c, d). Simultaneously, the levels of Yap1 were significantly upregulated by the overexpression of Yap1 (Fig. 6e). Flow cytometry results revealed that Yap1 overexpression significantly suppressed cell death (Annexin V-APC/7-AAD double positive cells) induced by a high concentration of TNF-α in hepatocytes (Fig. 6f, g). These results indicated that TNF-α functioned as an important factor in regulating the proliferation and cell death of hepatocytes via Yap1 expression and activity.

## Discussion

TNF-α is a double-edged sword for liver injury. Deletion of TNF-α or its receptor could alleviate LPS-induced liver injury but aggravate APAP-induced liver injury. In the present study, we showed that the concentration of TNF-α is critical for cell fate determination. High concentrations of TNF-α triggered hepatic cell death, but low concentrations of TNF-α could promote hepatic cell survival. We then discovered that the induction and activation of Yap is the key event in the determination of cell fate induced by TNF-α. These results provide new information for the therapeutic development of acute liver injury.

Studies have shown that liver injury caused by different factors has relatively specific inflammatory microenvironment characteristics^22,23^. We found that TNF-α was significantly increased in the livers of WT rats after LPS injection. Continuous LPS exposure lead to the activation of Kupffer cells, which resulted in the production of TNF-α. However, APAP-induced liver injury occurs in hepatocytes, which are the main metabolic cell of the human body^9,24^. APAP could be metabolized by hepatic cytochrome P450 to generate *N*-acetyl-p-benzoquinone imine (NAPQI). NAPQI is then detoxified by reacting with GSH. However, when hepatic GSH is exhausted, excessive NAPQI binds to cellular proteins covalently, which gives rise to mitochondrial dysfunction, oxidative stress, and ATP depletion^25,26^. The oxidative stress in turn leads to the nitration of mitochondrial proteins, the DNA damage of mitochondrial, ultimately resulting in the mitochondrial permeability transition and cell death^27,28^, which even lead to death of the rat. We found TNF-α expression did not increase in the early stage of APAP-induced liver injury. Therefore, the characteristics of TNF-α secretion were different between the two models. Our past research found that low doses of etanercept (a widely used inhibitor of TNF-α) alleviated CCl_4_-induced liver injury, but high doses of etanercept aggravated CCl_4_-induced liver injury^29^, which indicated that the function of TNF-α is concentration-dependent. We further elucidated the effect of TNF-α concentration on its functions. We injected different concentrations of TNF-α into TNF-α^−/−^ rats. The results showed that a low concentration of TNF-α could reduce the plasma AST and ALT contents in TNF-α^−/−^ rats and could promote the proliferation of BRL cells. However, a high concentration of TNF-α could increase plasma ALT and AST levels in TNF-α^−/−^ rats and even result in death^30^. In addition, the cell death rate of BRL cells was increased at a high concentration of TNF-α. LPS was found to stimulate the secretion of TNF-α in a dose-dependent manner in rats (Fig. S4A). We confirmed that noninjurious doses of LPS could ameliorate APAP-induced acute liver injury^31^, and only an optimal dose of LPS (0.25 mg/kg and 0.5 mg/kg) pretreatment could alleviate APAP-induced liver injury. However, LPS pretreatment with too low or too high dose could not alleviate APAP-induced liver injury (Fig. S4B). We showed previously that TNF-α was required for LPS-induced hepatoprotection against APAP-induced hepatotoxicity, as rats lacking TNF-α were not protected from liver injury by LPS preconditioning^31^. In brief, a moderate concentration of TNF-α induced by LPS could alleviate APAP-induced liver injury. These data suggested that the concentration of TNF-α is a very important factor influencing its functions. Attention should be paid to the level of TNF-α secretion upon liver injury. It was showed that systemic TNF-α in response to LPS was produced mainly by macrophages and neutrophils^8^. Deletion of liver macrophages using GdCl3 could protect rats from LPS-induced liver injury. These results indicated that blocking the TNF-α/TNFR1 pathway or reducing TNF-α secretion of immune cells maybe useful for disease progression when TNF-α secretion increases sharply, such as the early phase of immunological liver injury. However, downregulated TNF-α levels are not a good choice for treatment when TNF-α is maintained at the physiological level, for instance, in the early phase of APAP-induced liver injury. Previous studies have investigated the feasibility of TNF-α inhibitors in the management of liver injury. However, most of these trials failed to achieve a desired result^32–35^. The overinhibition of TNF-α using blocking agents might contribute to the failure of these trials.

Besides the concentration, the source of TNF-α is also a major concern. TNF-α could be produced by many different cells including Kupffer cells, lymphocytes, neutrophils, endothelial, glial cells, monocytes, etc. It have been showed that TNF-α from different cell sources have different functions. For instance, macrophage/neutrophil-derived and T-cell-derived TNF-α cannot perform functions of other cellular sources of TNF-α, and they have different functions in the promotion of autoimmune hepatitis^8^. In addition, Bonnardel et al.^36^ proved TNF-α released by dying Kupffer cells could activate stellate cells and endothelial cells. Piliponsky^37^ also showed TNF-α from basophils could enhance the innate immune response against bacterial infection. Further experiments required to explore the function of TNF-α from different sources in two types of liver injury.

TNFR1 is expressed on nearly all cells^38,39^. The binding of TNF to TNFR1 can induce either cell survival or different forms of cell death^40^. It has been shown that the engagement of TNF-α with TNFR1 activates cell survival and proliferation pathways if complex 1 is retained on the cell membrane^41^. Complex 1 leads to canonical (classical) NF-κB activation, which translocates to the nucleus and activates the transcription of genes that regulate cell survival and proliferation. In addition, TNFR1 is classified as a death receptor, and classical apoptosis was shown to occur upon TNF binding to TNFR1. Therefore, TNF-α/TNFR1 signal transduction is a constant balancing act between these opposing functions^42^. We confirmed that the protective or damaging effects of TNF-α were mediated via TNFR1 in liver injury caused by different factors. The molecular mechanisms underlying the roles of the TNF-α/TNFR1 pathway in cell fate determination may contribute to developing pharmacological therapies for liver injury.

Hippo-Yap plays important roles in regulating cell proliferation/death homeostasis and mediating tissue development or organ size^43^. It has been shown that Yap translocates to the nucleus and is activated in response to TNF-α in endothelial cells^44^. Gao et al.^45^ confirmed that TNF-α triggers IKK-mediated Yap activation in breast cancer cells. Other studies have suggested that TNF-α could phosphorylate Yap by inducing LATS2 expression in oral squamous cell carcinoma^46^. TNF receptor-associated factor 6 (TRAF6), which is a member of the TNF receptor-associated factor family downstream of the TNF receptor, could regulate Yap signaling by promoting the ubiquitination and degradation of MST1 in pancreatic cancer^47^. These findings suggest that TNF-α could affect Yap activity. Here, we determined that a low concentration of TNF-α induced Yap1 expression, while knockdown of Yap1 in BRL cells significantly inhibited cell proliferation and enhanced cell death. However, a high concentration of TNF-α induced the phosphorylation and inactivation of Yap1, preventing its nuclear import. Overexpression of Yap1 by a virus vector significantly decreased cell death of BRL cells. Taken together, these results indicate that Yap plays an important role in directing cell fate (Fig. 6h).

In conclusion, we have provided novel evidence for the function of the TNF-α/TNFR1 pathway in liver injury and uncovered the critical role of Yap activity in the determination of cell fate induced by different concentrations of TNF-α, which may provide a potential therapeutic strategy for liver injury.

## Materials and methods

### Animal model and treatment

Male Sprague–Dawley rats (6–8 weeks old, weighing 160–180 g) were purchased from the Shanghai Experimental Animal Center of the Chinese Academy of Sciences (Shanghai, China). TNF-α knockout (TNF-α^−/−^) rats, TNFRSF1A knockout (TNFR1^−/−^) rats, and TNFRSF1B knockout (TNFR2^−/−^) rats were all established by Nanjing Xunqi Biotechnology Co. Ltd. by CRISPR/Cas9-based genome editing^48^. Exon 1 of the TNF-α gene was targeted to induce sequence deletions with frame-shifts, which were identified by PCR genotyping and sequence analysis. Breeding of TNF-α^+/−^ rats was done to generate TNF-α^−/−^ rats for experiments. DNA sequencing shows a 5-bp deletion in exon1 of TNF-α gene in the TNF-α^−/−^ rat (Fig. S1A). TNFR1^−/−^ rats and TNFR2^−/−^ rats were constructed using the same approach. DNA sequencing shows a 34-bp deletion in exon 2 of TNFR1 gene in the TNFR1^−/−^ rat (Fig. S1B) and a 2-bp deletion in exon 3 of TNFR2 gene in the TNFR2^−/−^ rat (Fig. S1C). All animals were maintained at the Laboratory Animal Center of the Second Military Medical University. The experimental protocols were approved by the Institutional Animal Care and Use Committee of the Second Military Medical University. Some rats were administered freshly prepared APAP (Sigma, MO, USA) at 1 g/kg or LPS (Sigma) at 10 mg/kg intraperitoneally to evaluate hepatotoxicity and monitor mortality at 72 h. Peripheral blood and liver tissue samples were collected at 0, 6, or 12 h after LPS or APAP injection. Some TNF-α^−/−^ rats were injected with different concentrations of TNF-α (R&D Systems, MN, USA), and the plasma was collected at 12 h after the injection. GdCl_3_⋅6H_2_O (10 mg/kg, Sigma) given at 24 h before LPS administration was used to inhibit Kupffer cells. In some experiments, rats were treated with different doses of LPS (0.05, 0.25, 0.5, 2.5, 5 mg/kg) or saline intraperitoneally 24 h before performing the APAP-induced liver injury model. The rats were randomly assigned to interventions.

### Measurement of liver function

Alanine aminotranferease (ALT) and aspartate aminotransferase (AST) are enzymes located in liver cells, their levels have been regarded as markers of liver injury^49^. The levels of AST and ALT were measured using an autoanalyzer (Spotchem Co., Kyoto, Japan).

### Hematoxylin–eosin staining of liver sections

Liver tissues were fixed in 10% neutral buffered formalin and embedded in paraffin. Sections (4 μm) were stained with hematoxylin and eosin (H&E) following standard protocols and subsequently analyzed under a light microscope.

### Terminal dUTP nick-end labeling staining

Apoptotic cells were detected using an in situ cell detection kit (Roche, Mannheim, Germany) according to the manufacturer’s instructions. The sections were visualized using standard fluorescence microscopic techniques.

### Measurement of reactive oxygen species (ROS)

The levels of hepatic glutathione (GSH) and the lipid peroxidation product malondialdehyde (MDA) were detected by using commercial kits according to the manufacturer’s protocols (Nanjing Jiancheng Bioengineering Institute, Jiangsu, China).

### Detection of TNF-α

TNF-α in the liver was measured using Rat Inflammation Array Q1 (RayBiotech, QAR-INF-1, GA, USA) according to the manufacturer’s instructions. The levels of TNF-α in plasma were determined using an ELISA Kit (R&D Systems, MN, USA) according to the manufacturer’s instructions.

### Cell culture

Buffalo rat liver cells (BRL cells) were purchased from Cell Bank at the Chinese Academy of Sciences (Shanghai, China). Cells were cultured in DMEM containing 10% FBS in a humidified incubator at 37 °C and 5% CO_2_. BRL cells were planted in 96-well plates or 6-well plates and treated with various concentrations of TNF-α (0, 10, and 500 ng/mL; R&D Systems, MN, USA) in DMEM without serum for 6 h.

### siRNA and adenovirus transfection

Small interfering RNA against TNFR1 was synthesized by Biomics Biotechnologies Co. Ltd. (Nantong, China). The recombinant adenoviruses carrying short hairpin RNA (shRNA) plasmids against Yap1 and the pAdeno vector containing the wild-type Yap1 insert were provided by OBiO Technology Corp (Shanghai, China). BRL cells were seeded in 6-well plates at a density of 2 × 10^5^ cells/well in antibiotic-free normal growth medium supplemented with FBS. BRL cells were transfected with siRNA-TNFR1 or adenovirus plasmid using FuGENE HD according to the manufacturer’s protocol. Western blotting was performed to detect protein levels 12 h post transfection.

### Cell viability

Cell viability was tested 6 h after treatment with different concentrations of TNF-α by a Cell Counting Kit-8 (CCK-8, 10 μL/well), and the absorbance at 450 nm was measured after 1 h of incubation at 37 °C.

### Cell death assay

Cell death analysis was performed 6 h after treatment with different concentrations of TNF-α using the FITC Annexin V Apoptosis Detection Kit (BD Biosciences, CA, USA) according to the manufacturer’s protocols. All samples were analyzed on a FACSCalibur flow cytometer (BD Biosciences, CA, USA).

### Western blotting

Cytoplasmic and nuclear fraction separation was performed using NE-PER nuclear and cytoplasmic extraction reagents (Thermo, MA, USA). Total cellular proteins were collected using RIPA lysis buffer (Beyotime, Jiangsu, China). Equal amounts of protein were electrophoresed by sodium dodecyl sulfate-polyacrylamide gel electrophoresis (SDS-PAGE). A wet transfer method was used to electrophoretically transfer proteins from the native gel to nitrocellulose membranes, which were then probed with anti-p-LATS1/2^T1079/1041^ (1:1000, Sigma, SAB4504615), anti-LATS1 (1:1000, Sigma, SAB1300096), anti-p-MST1/2 (1:1000, Sigma, SAB4504042), anti-MST1 (1:1000, Abcam, AB124787, Cambridge, UK), anti-Yap1 (1:1000, Abcam, AB39361), anti-p-Yap^S127^ (1:1000, Abcam, AB76252) antibodies, and developed with the BeyoECL Plus substrate system (Beyotime). The blots were stripped and reprobed with GAPDH antibody (1:2000, Bioworld, AP0063, CA, USA) to confirm equal protein loading.

### Immunohistochemistry

Paraffin-embedded liver tissues were cut into 4 μm serial sections. Immunohistochemistry (IHC) was performed using anti-CD68 (1:200, Abcam, AB31630). The detailed method has been published previously^31^.

### Immunofluorescence

Cells were cultured on glass coverslips, fixed with 4% paraformaldehyde, permeabilized in PBS containing 0.4% Triton X-100, and blocked with 1% bovine serum albumin (BSA). Cells were then incubated with anti-Yap1 (1:200, Abcam, AB39361) overnight at 4 °C. The glass coverslips were washed with PBS and incubated with a fluorescent secondary antibody for 1 h at 37 °C. Then, the glass coverslips were washed with PBS and stained with DAPI. Images of the sections were obtained using Image-Pro Plus 4.5 software (Media Cybernetics, Silver Spring, MD, USA).

### Statistical analysis

Survival curves were calculated by the Kaplan-Meier method, and differences in survival rates were compared by using the log-rank test. Statistical differences were assessed using one-way analysis of variance followed by Bonferroni post-hoc test. A *p*-value < 0.05 was considered statistically significant. Quantitative data are presented as the mean ± SD.

## Supplementary information

Supplementary Figure 1

Supplementary Figure 2

Supplementary Figure 3

Supplementary Figure 4

Supplementary Figure Lgends
