# Supplementary figures and images for: The concentration of tumor necrosis factor-α determines its protective or damaging effect on liver injury by regulating Yap activity
Source: Cell Death Dis. 2020 Jan 27;11(1):70. doi: 10.1038/s41419-020-2264-z (PMC6985193; doi:10.1038/s41419-020-2264-z)

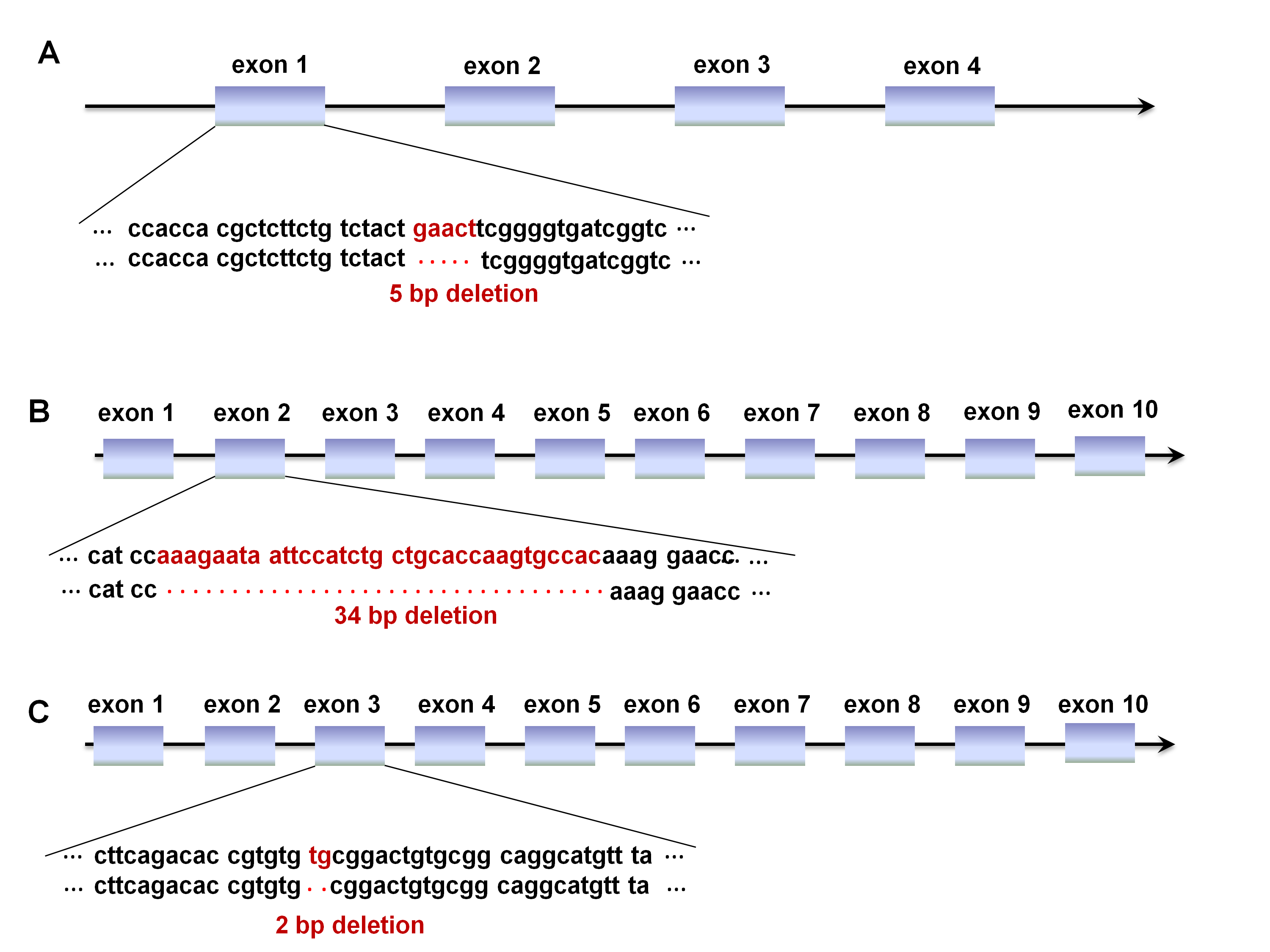

Supplement: Supplementary file 1 — Supplementary Figure 1 [file 41419_2020_2264_MOESM1_ESM.tif]

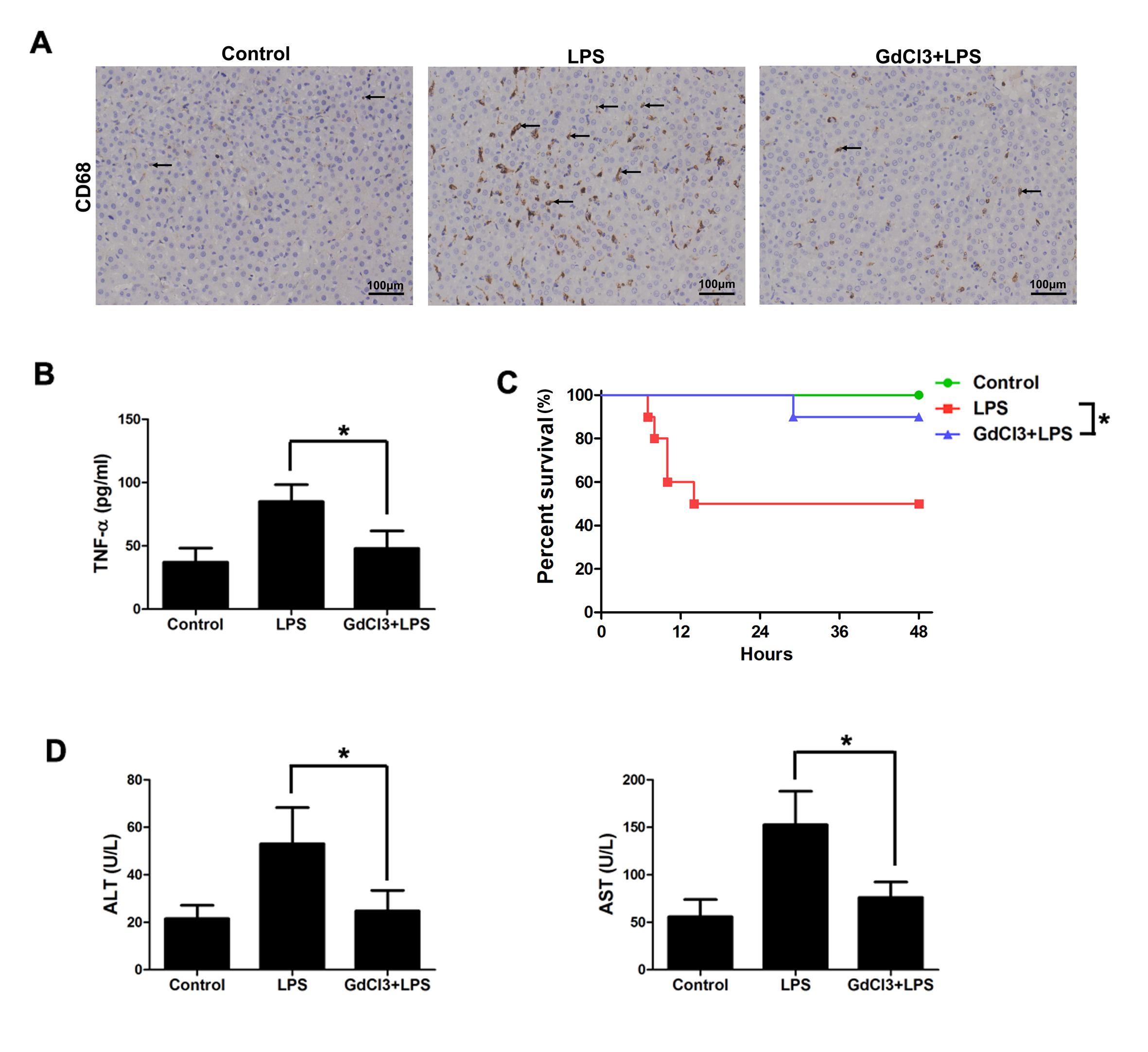

Supplement: Supplementary file 2 — Supplementary Figure 2 [file 41419_2020_2264_MOESM2_ESM.tif]

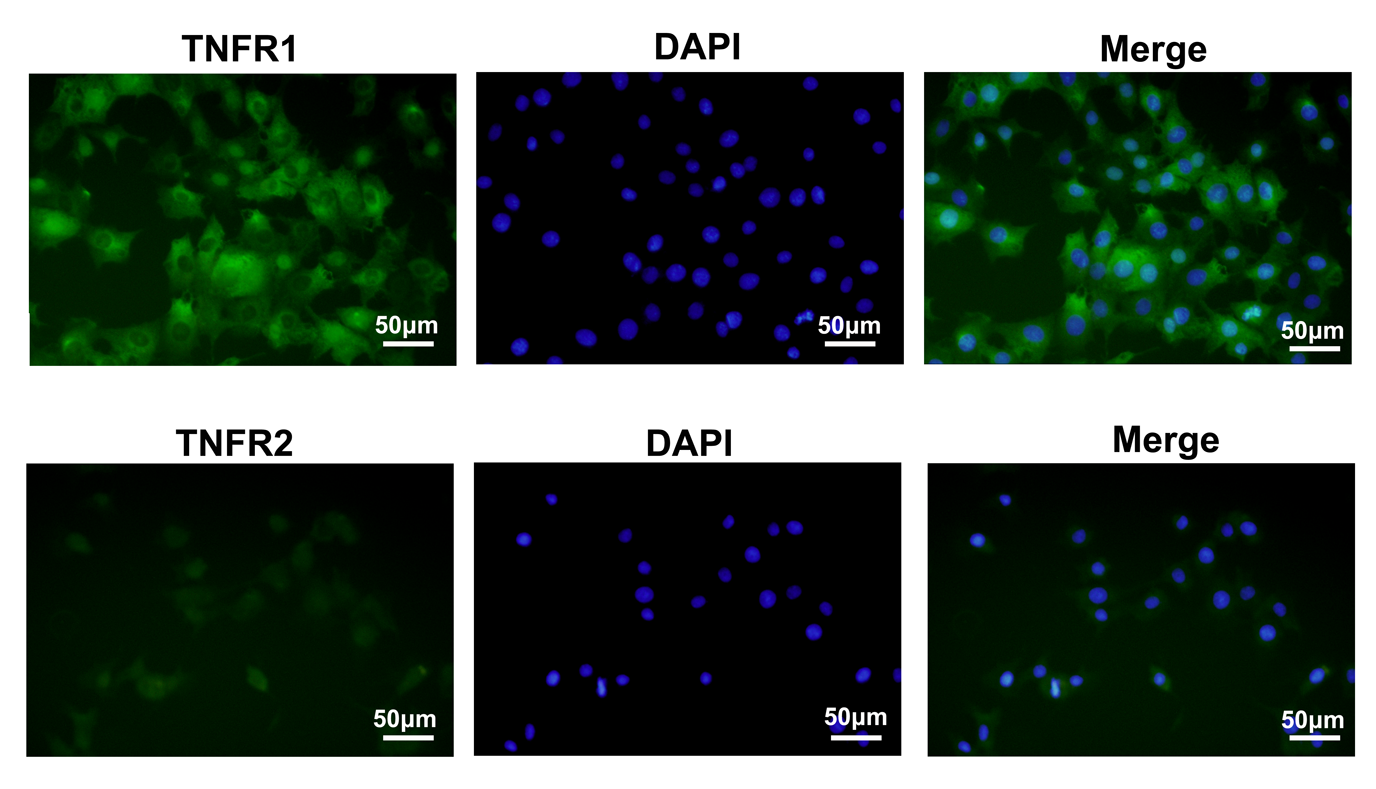

Supplement: Supplementary file 3 — Supplementary Figure 3 [file 41419_2020_2264_MOESM3_ESM.tif]

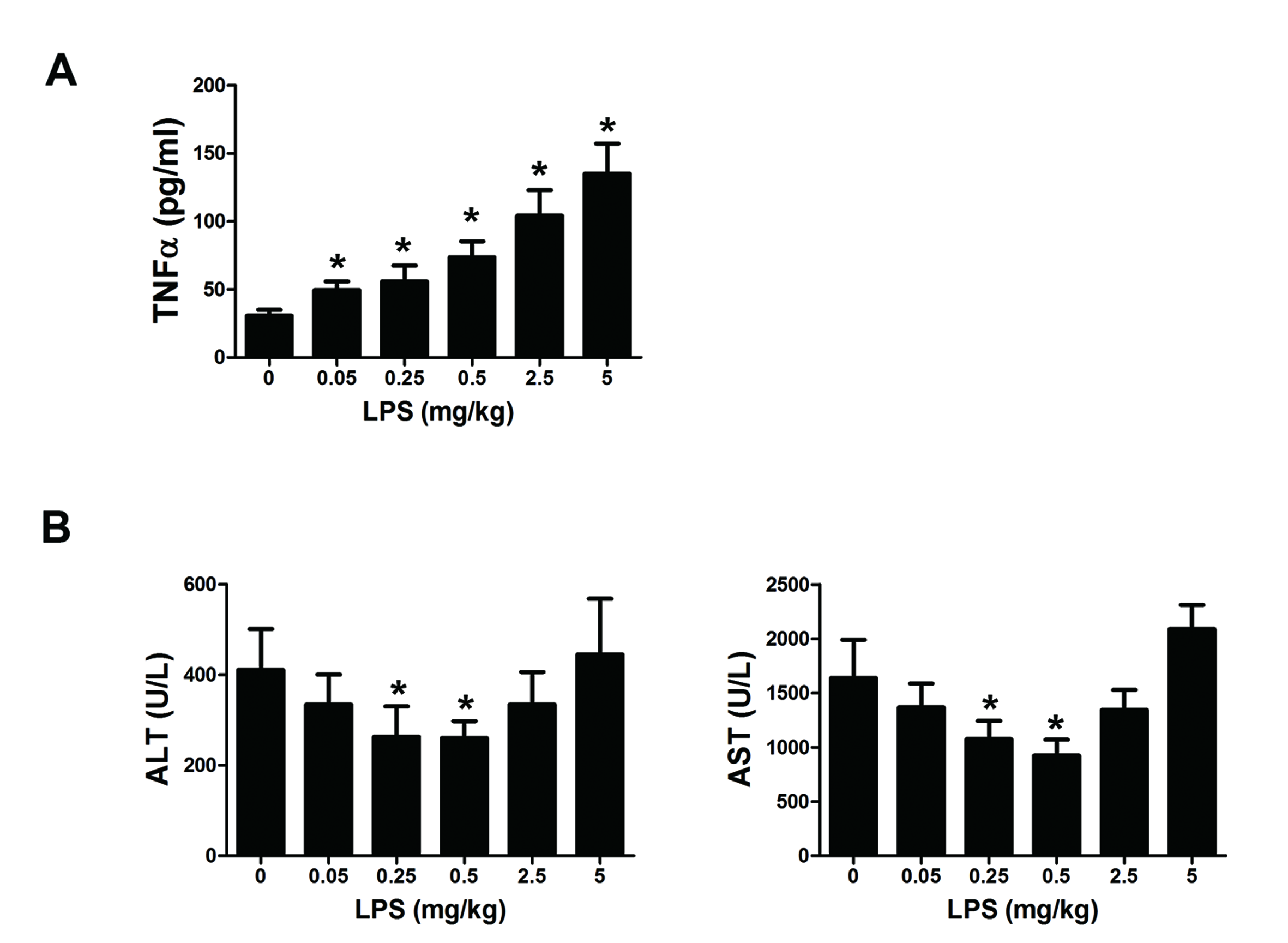

Supplement: Supplementary file 4 — Supplementary Figure 4 [file 41419_2020_2264_MOESM4_ESM.tif]
