# Supplementary material for: The concentration of tumor necrosis factor-α determines its protective or damaging effect on liver injury by regulating Yap activity
Source: Cell Death Dis. 2020 Jan 27;11(1):70. doi: 10.1038/s41419-020-2264-z (PMC6985193; doi:10.1038/s41419-020-2264-z)
Supplement: Supplementary file 5 — Supplementary Figure Lgends [file 41419_2020_2264_MOESM5_ESM.docx]

**Supplementary Figure Legends**

**Figure S1. Sequencing results of (A) TNF-α^-/-^, (B) TNFR1^-/-^ and (C) TNFR2^-/-^.** Exons marked with box. “. ” indicates nucleotide deletion.

**Figure S2. Inhibition of TNF-α production by Kupffer cells could alleviate LPS-induced liver injury.** GdCl_3_ (10 mg/kg) was injected intravenously 24 hours before LPS administration. (A) Representative images of CD68 staining in the livers of LPS- or GdCl_3_+LPS-treated rats. Four liver sections were randomly selected from each group and used for immunohistochemistry analysis. Arrow indicates the CD 68^+^ Kupffer cells. (B) Expression of TNF-α was detected in plasma at 6 hours after LPS intraperitoneal injection. (C) The mortality rate of rats induced by GdCl_3_ pretreated with or without LPS (n=10 for each group). (D) The levels of ALT or AST at 6 hours after LPS injection (n=4 for each group). Data are expressed as the mean ± SD. *p<0.05.

**Figure S3. The location of TNFR1 and TNFR2 were expressed in BRL cells.** TNFR1 and TNFR2 antibodies were used for immunofluorescence staining. Bar: 50μm

**Figure S4. Pretreatment with the appropriate doses of LPS provided protection against subsequent challenge with APAP.** Rats were intraperitoneally injected with LPS or saline at 24 hours before performing the APAP-induced liver injury model. (A) The concentration of plasma TNF-α in WT rats injected with different doses of LPS for 24 hours (n=4 for each group). (B) The levels of ALT or AST in the plasma of WT rats at 0 and 12 hours after APAP injection (n=4 for each group). Data are expressed as the mean ± SD. *p<0.05 versus the control group.
